# Neighborhood Social Determinants of Triple Negative Breast Cancer

**DOI:** 10.3389/fpubh.2019.00018

**Published:** 2019-02-18

**Authors:** Fokhrul Hossain, Denise Danos, Om Prakash, Aubrey Gilliland, Tekeda F. Ferguson, Neal Simonsen, Claudia Leonardi, Qingzhao Yu, Xiao-Cheng Wu, Lucio Miele, Richard Scribner

**Affiliations:** ^1^School of Medicine, Louisiana State University Health Sciences Center, New Orleans, LA, United States; ^2^School of Public Health, Louisiana State University Health Sciences Center, New Orleans, LA, United States; ^3^Louisiana State University Health Sciences Center, New Orleans, LA, United States

**Keywords:** racial disparity, triple negative breast cancer, SEER database, multi-level modeling, concentrated disadvantage, socioeconomic factors

## Abstract

Triple Negative Breast Cancer (TNBC) is an aggressive, heterogeneous subtype of breast cancer, which is more frequently diagnosed in African American (AA) women than in European American (EA) women. The purpose of this study is to investigate the role of social determinants in racial disparities in TNBC. Data on Louisiana TNBC patients diagnosed in 2010–2012 were collected and geocoded to census tract of residence at diagnosis by the Louisiana Tumor Registry. Using multilevel statistical models, we analyzed the role of neighborhood concentrated disadvantage index (CDI), a robust measure of physical and social environment, in racial disparities in TNBC incidence, stage at diagnosis, and stage-specific survival for the study population. Controlling for age, we found that AA women had a 2.21 times the incidence of TNBC incidence compared to EA women. Interestingly, the incidence of TNBC was independent of neighborhood CDI and adjusting for neighborhood environment did not impact the observed racial disparity. AA women were more likely to be diagnosed at later stages and CDI was associated with more advanced stages of TNBC at diagnosis. CDI was also significantly associated with poorer stage-specific survival. Overall, our results suggest that neighborhood disadvantage contributes to racial disparities in stage at diagnosis and survival among TNBC patients, but not to disparities in incidence of the disease. Further research is needed to determine the mechanisms through which social determinants affect the promotion and progression of this disease and guide efforts to improve overall survival.

## Introduction

Understanding racial disparities in breast cancer is complicated by the fact disparities vary among the different breast cancer subtypes. Characterizing the social inequalities that give rise to each of these disparities is necessary to inform preventive interventions and work toward health equity (1–4). This is especially true of triple negative breast cancer (TNBC) due to the aggressive nature of this type of cancer and relative lack of treatment modalities available to patients.

TNBC is an aggressive, heterogeneous subtype of breast cancer, immunohistochemically negative for estrogen receptor α (ER^−^) and progesterone receptor (PR^−^), as well as lacking amplification of the human epidermal growth factor receptor 2 locus (HER2^−^). While TNBC accounts for an estimated 12–15% of all breast cancers, the unique epidemiology and biology of TNBC draw substantial research attention. Numerous studies have shown that relative to European Americans (EA/White), TNBC is significantly more prevalent among African American (AA/Black) women (5–8), and is associated with a poorer prognosis than other subtypes of breast cancer (9, 10). TNBC is characterized by diagnosis at a younger age, higher tumor grade, and larger tumor size (11, 12).

Distinct racial disparities exist in TNBC outcomes (i.e., incidence, stage at diagnosis, and survival). For this reason, there is a vital need to understand the role of biological and non-biological factors in disparities (4, 13–15). Some studies have begun to describe the nature of these racial disparities in terms of the risk due to non-biological social determinants (4, 7, 16, 17). However, the findings are not yet at the point they can establish the translational impact necessary for developing policy interventions.

Several issues concerning TNBC disparities research need to be addressed to draw translationally impactful conclusions. First, there needs to be an appreciation of the fact that the various outcomes (e.g., incidence, stage at diagnosis, survival) involve different stages in cancer carcinogenesis (i.e., initiation, promotion and progression). At each of these stages unique factors are likely to contribute to racial disparities, which may or may not include social determinants (18). Consequently, results indicating a role for social determinants in explaining a TNBC disparity may vary by the stage of carcinogenesis. Second, characterizing the role of modifiable factors in the physical and social environment requires the integration of secondary data to characterize the local environment (i.e., neighborhood) in which the patient lives. This type of integration requires an analytic approach involving large population-based datasets using multilevel models at the appropriate scale to characterize social determinants (3, 19).

In both national and state level registry based studies, AA women have a 2-fold increase in risk of being diagnosed with TNBC compared with EA (13, 15). Several studies have incorporated measures of socioeconomic risk in individual level models and found these factors appeared unrelated to incidence and therefore did not explain any of the racial disparity (7, 16, 17). Racial disparities in TNBC stage at diagnosis and survival also exist, with AA women more often diagnosed at later stages and experiencing lower survival (20). In contrast to TNBC incidence, racial disparities in TNBC stage at diagnosis and survival do appear to be partially, if not completely, socially determined. Tao et al. (4) found measures of neighborhood SES to fully explain the disparity in stage-specific survival for AA women diagnosed with TNBC. These findings suggest the etiologic pathways for TNBC disparities vary across the continuum of the disease.

Besides the District of Columbia, Louisiana ranks first in breast cancer mortality (21). Unfortunately, the excess breast cancer mortality can be almost entirely attributed to the high mortality rate for AA women. Among AA women, the mortality rate is 15% above the national average, while among EA women, the mortality rate is comparable to the national average (21). Racial disparities in breast cancer mortality are complex and originate from a multitude of factors including greater incidence of more aggressive subtypes, such as TNBC among AAs, later stages at diagnosis, higher levels of comorbidities, as well as inequality in treatment, access to care, and adherence (22–25). In this study, we conducted an analysis of TNBC incidence, stage at diagnosis, and survival in Louisiana to better understand the origins of racial disparities in this disease by assessing the role of a robust measure of risk associated with the neighborhood environment in explaining racial disparities across TNBC outcomes in the same population. In addition, we introduce a multilevel approach to the analysis of tumor registry data that permits inferences regarding the potential role of neighborhood level social determinants with respect to individual level risk (26, 27).

## Methods

### Case Definition

This study used 2010–2012 data from the Louisiana Tumor Registry (LTR), a participant of the National Cancer Institute's (NCI) Surveillance, Epidemiology and End Results (SEER) Program and the Centers of Disease Control and Prevention's National Program of Cancer Registries (CDC-NPCR). All study variables regarding demographics (age, sex, race, ethnicity, address) and tumor characteristics (site, behavior, histology, stage, grade) were collected from hospital and medical records in accordance with registry guidelines. Primary invasive breast cancer cases were identified by International Classification of Diseases for Oncology, Third Edition (ICDO-3) site codes C500-C509. ICD-O-3 histology codes 9050-9055, 9140, and 9590-9989 were excluded. *In-situ* cases were also excluded. Breast cancer cases were classified into subtypes by estrogen receptor, progesterone receptor and human epidermal growth factor 2 (HER2) status, which have been routinely collected by SEER registries since 2010 (28). Hormone receptor (HR) status was considered negative if the tumor lacked both estrogen and progesterone reactivity. The four molecular breast cancer subtypes were defined as HR+/HER–, HR+/HER+, HR–/HER2– (Triple Negative), and HR–/HER2+. The Louisiana State University Health Sciences Center Institutional Review Board approved this research.

### Geocoding Cases

TNBC cases were geocoded to 2010 census tracts using the Automated Geospatial Geocoding Interface Environment system, which was developed as a uniform geocoding platform for open use by cancer registries^1^. To ensure a high certainty of patient location, we restricted matched cases to those geocoded using street address at time of diagnosis. We used 2010 US Census population for demographic groups within census tracts as the population at-risk in determining incidence of disease. Census tract socioeconomic measures were obtained from the US Census American Community Survey (ACS).

### Measuring Disadvantage

Neighborhood concentrated disadvantage index (CDI) scores for census tracts were calculated based on the PhenX Toolkit Protocol^2^. The toolkit is an established resource of consensus measures of phenotypes and exposures for biomedical research. CDI is a sample-based composite score derived from a principal components analysis of 6 measures at the census tract level (given as percentages): (1) individuals below the poverty line; (2) households receiving public assistance income; (3) female-headed families; (4) individuals that are unemployed; (5) individuals below the age of 18; and (6) individuals that are Black. The construct operationalizes urban theory regarding the overconcentration of Blacks, children and female-headed families in economically disadvantaged neighborhoods (29). This measure of disadvantage has been associated with poor outcomes in breast and ovarian cancer (30, 31). We derived CDI using 2008–2012 5-year estimates of American Community Survey (ACS), to best align with the study period^3^. All measures follow US Census definitions provided by the ACS. Factor scores for study census tracts follow a standard normal distribution with a mean of zero and standard deviation of 1. Factor loadings are provided as Supplementary Table 1.

### Census Tract Exclusions

From the 2010 US Census, Louisiana has 1,148 census tracts. Standard US census tracts typically contain between 2,500 and 8,000 residents and are designed to be relatively homogenous in terms of socioeconomic characteristics^4^. Census tracts with zero population per the 2010 US census were not included (*n* = 19). We excluded eight low-population (population of 500 or less) census tracts and the census tract constituting the Orleans Parish Prison. After these exclusions, the study included 1,120 census tracts. Cancer outcomes were assessed for women aged 30 or older, of Black or White race, who resided in an eligible Louisiana census tract during the study period. Cases in women younger than 30 years old and cases in races other than black or white were not included in these analyses due to small age and race group numbers. Inclusion of Hispanic women was based on identified race regardless of ethnicity.

### Outcome Determinations

Incidence of disease was a Binomial response constructed from the number of incident TNBC cases out of the population-years at risk for age and race specific groups within census tracts. Stage at diagnosis was a Multinomial response based on the coded SEER summary 2000 system, with 3 stages, local, regional and distant^5^. Patient survival was calculated as the number of months elapsed from date of diagnosis to date of last contact or death, as recorded by the LTR. The latest date of contact in the patient dataset was August 2016. The event of interest was breast-cancer specific survival and was determined using patient vital status and cause of death, based on SEER cause-specific death coding system^6^.

### Statistical Analysis

To examine the role of neighborhood disadvantage in TNBC outcomes we used multivariable multilevel models of individuals (level 1) nested within census tracts (level 2). A random intercept at the census tract level was used to account for correlation among individuals within census tracts, or neighborhood clustering, for all models. Cancer incidence was modeled with binomial regression. Stage at diagnosis was analyzed using proportional odds logistic regression. We compared stage-specific survival for Black and White races using Kaplan-Meier plots of time to breast cancer death. Stage-specific survival was analyzed using multilevel Cox proportional hazards regression (frailty model) and included fixed effects for stage at diagnosis. For the study, we assumed that racial disparities in TNBC are independent of age (4, 7). Initial multilevel models were executed as mixed effects models with fixed effects for age, as 10-year age groups, and race. This model was used to estimate racial disparities in TNBC incidence and outcomes after adjusting for the effects of age. To test the hypothesis that neighborhood concentrated disadvantage is associated with adverse outcomes among both races, a subsequent model included a fixed effect for CDI, in addition to main effects for age and race. All statistical analyses were performed in SAS version 9.4 software (SAS Institute, Cary, NC). Generalized mixed linear models were executed using the GLIMMIX Procedure, with maximum likelihood estimation via adaptive quadrature method. Cox proportional hazards regression was executed in the PHREG Procedure. We considered two-sided *p* < 0.05 as statistically significant.

## Results

Breast cancer cases diagnosed in Louisiana from 2010 to 2012 with a histologically confirmed subtype are summarized in Table 1. TNBC cases that met the study criteria accounted for 14.79% (*n* = 1,216) of cases. The demographic distribution of cases varied by subtype. An earlier age of onset for TNBC was observed in the patient sample. More than 25% of TNBC cases were diagnosed in women younger than 50 years old. Racial distribution of breast cancer cases also differed by subtypes. AA women constituted 47% of TNBC cases, the highest proportion for any subtype. There were stark differences in tumor grade and stage at diagnosis by subtype, with HR+/HER2– tumors having a greater share of low grade and localized tumors compared to other subtypes.

**Table 1:** Demographics and tumor stage characteristics of breast cancer patients by molecular subtype, Louisiana 2010–2012.

	**HR+/HER2−**	**HR+/HER2+**	**HR−/HER2+**	**TNBC**
N	5,650	944	410	1,216
**AGE, YEARS (%)**				
30–39	2.81	5.51	8.05	7.32
40–49	13.27	17.16	17.80	18.59
50–59	23.86	24.79	27.07	29.28
60–69	27.65	25.53	26.34	23.52
70+	32.41	27.01	20.73	21.30
**RACE (%)**				
White	73.82	69.70	61.46	51.81
Black	25.33	29.77	37.56	47.45
Other	0.85	0.53	0.98	0.74
**SEER SUMMARY STAGE 2000 (%)**^**a**^				
Local	64.12	56.04	51.95	57.81
Regional	30.48	36.12	37.32	33.31
Distant	5.12	7.73	10.49	8.39
Unknown	0.28	0.11	0.24	0.49
**CDI**				
Mean (Std Dev)	−0.21 (0.88)	−0.16 (0.9)	−0.04 (0.91)	0.00 (0.97)

*(N = 8,220). HR, Hormone Receptor; HER2, Human Epidermal Growth Factor 2; TNBC, Triple Negative Breast Cancer; SEER, Surveillance, Epidemiology and End Results Program; CDI, Concentrated Disadvantage Index*.

a https://www.registrypartners.com/seer-summary-staging-manual-2000/

### Incidence

**Figure 1:**
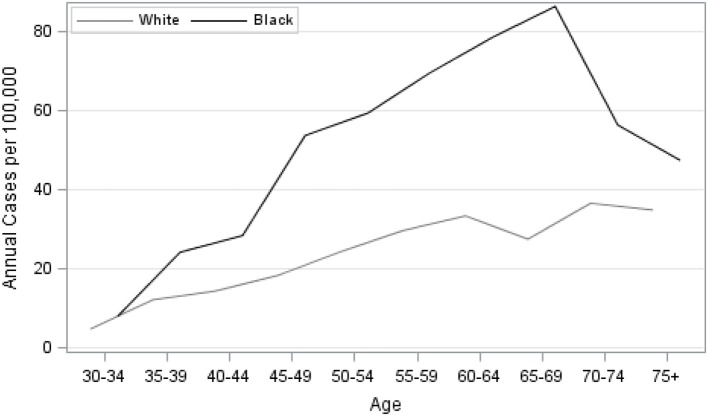
Age-specific unadjusted incidence of triple-negative breast cancer among females by race, Louisiana 2010–2012.

**Figure 2:**
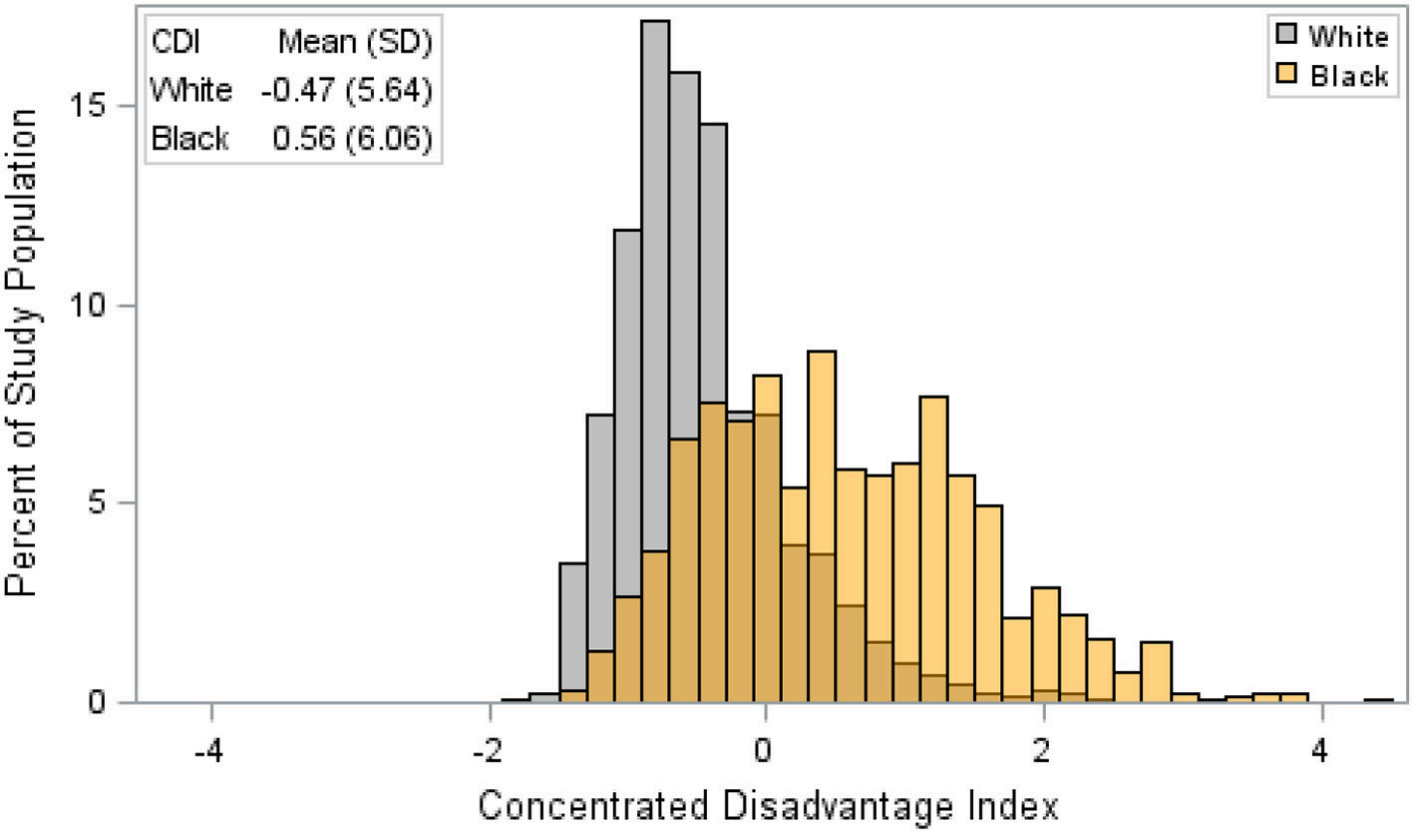
Distribution of concentrated disadvantage index among female triple negative breast cancer patients by race, Louisiana 2010–2012. CDI is a sample-based index, where scores have a mean of 0 and a standard deviation of 1. Therefore, a single unit increase in CDI represents a one standard deviation increase in neighborhood disadvantage.

Our analysis of TNBC incidence included 1,308,564 female residents aged 30 years and older. AA women comprised 30.9% of the study population. AA women had significantly higher age-specific incidence of TNBC, given as cases per 100,000, compared to EA women (Figure 1). There was also a notable difference in the exposure to CDI for Louisiana women by race. Figure 2 illustrates that AA women were disproportionately represented in more disadvantaged areas, with an average CDI of 0.56 (standard deviation *SD* = 6.06) compared to −0.47 (*SD* = 5.64) among EA women. Table 2 provides adjusted risk ratios (RR) and 95% confidence intervals (CI) from multilevel binomial regression for triple negative breast cancer incidence. In our initial model, controlling for age, AA women in Louisiana had 2.21 times the risk of TNBC compared to EA [*RR* = 2.21, 95% CI (1.96, 2.48)]. In model 2, neighborhood concentrated disadvantage was not significantly associated with incidence of the disease [*RR* = 0.96, 95% CI (0.89, 1.03)] and the racial disparity appeared to be independent of CDI [*RR* = 2.30, 95% CI 2.01, 2.64)].

**Table 2:** Estimated risk ratios (RR) and 95% confidence intervals (CI) from multilevel binomial regression of triple negative breast cancer, Louisiana 2010–2012.

	**Model 1**	**Model 2**
	**RR (95% CI)**	**RR (95% CI)**
**AGE, YEARS**		
30–39 (reference)	1.00	1.00
40–49	2.33 (1.82, 2.99)	2.33 (1.82, 2.99)
50–59	3.68 (2.91, 4.66)	3.69 (2.92, 4.66)
60–69	4.44 (3.49, 5.65)	4.45 (3.50, 5.66)
70+	4.06 (3.18, 5.18)	4.07 (3.19, 5.20)
**RACE**		
White (reference)	1.00	1.00
Black	2.21 (1.96, 2.48)	2.30 (2.01, 2.64)
**CDI**		
1 SD Increase	–	0.96 (0.89, 1.03)

*RR, risk ratio; CI, confidence interval; CDI, concentrated disadvantage index; SD, standard deviation*.

*Model 1 is adjusted for age and race. Model 2 is adjusted for age, race and CDI*.

*All models include random intercept for US census tracts*.

### Stage at Diagnosis

Our analysis of stage at diagnosis (local, regional, distant) included all TNBC patients in the sample with known stage (*n* = 1,201). Results from multilevel proportional odds logistic regression models are presented as Table 3. In our initial model, controlling for age, AA women had 42% greater odds of being diagnosed with TNBC at a later stage [*OR* = 1.42, 95% CI (1.11, 1.81)]. Results from model 2 showed a single standard deviation increase in CDI was associated with a 20% increase in the odds of diagnosis at a more advanced stage [*OR* = 1.20, 95% CI (1.03, 1.39)]. After adjusting for CDI, the racial disparity was no longer statistically significant [*OR* = 1.17, 95% CI (0.88, 1.56)].

**Table 3:** Estimated odds ratios (OR) and 95% confidence intervals (CI) from multilevel proportional odds logistic regression models of SEER stage at diagnosis in triple negative breast cancer, Louisiana 2010–2012.

	**Model 1**	**Model 2**
	**OR (95% CI)**	**OR (95% CI)**
**AGE, YEARS**		
30–39 (reference)	1.00	1.00
40–49	0.62 (0.38, 1.02)	0.62 (0.38, 1.01)
50–59	0.56 (0.35, 0.90)	0.56 (0.35, 0.89)
60–69	0.74 (0.46, 1.19)	0.72 (0.45, 1.15)
70+	0.66 (0.40, 1.07)	0.64 (0.39, 1.04)
**RACE**		
White (reference)	1.00	1.00
Black	1.42 (1.11, 1.81)	1.17 (0.88, 1.56)
**CDI**		
1 SD Increase		1.20 (1.03, 1.39)

*OR, odds ratio; CI, confidence interval; SEER, Surveillance, Epidemiology and End Results Program; CDI, concentrated disadvantage index; SD, standard deviation*.

*Model 1 is adjusted for age and race. Model 2 is adjusted for age, race and CDI*.

*All models include random intercept for US census tracts*.

### Survival

**Figure 3:**
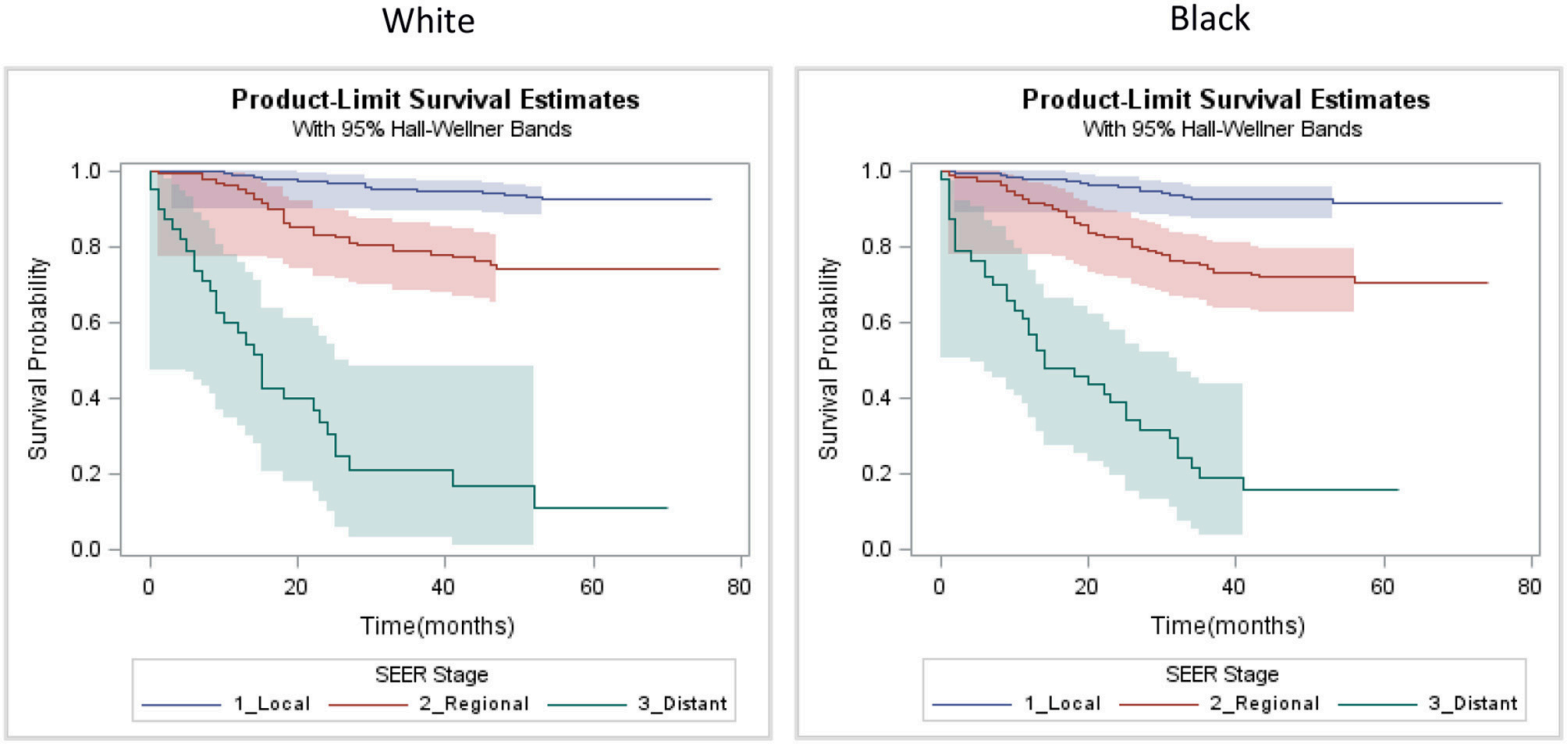
Kaplan-Meier SEER stage-specific survival plots of triple negative breast cancer patients by race, Louisiana 2010–2012.

Our analysis of survival included patients for which TNBC was their first cancer diagnosis and the tumor was of known stage (*n* = 989). In this sample, 191 patients experienced breast cancer-related death during follow up. Stage-specific survival curves (Figure 3) suggested similar survival among Black and White patients. Multilevel Cox proportional hazard model results are provided as Table 4. In our initial model, controlling for stage and age at diagnosis, we found no statistically significant difference in stage-specific survival by race [*HR* = 1.08, 95% CI (0.80, 1.46)]. However, when introduced into the model, CDI was significantly associated with poorer survival [*HR* = 1.19, 95% CI (1.01, 1.39)].

**Table 4:** Estimated hazard ratios (HR) and 95% confidence intervals (CI) from Cox proportional hazards model of cancer-related death in triple negative breast cancer, Louisiana 2010–2012.

	**Model 1**	**Model 2**
	**HR (95% CI)**	**HR (95% CI)**
**SEER SUMMARY STAGE 2000**^**a**^		
Local (reference)	1.00	1.00
Regional	4.03 (2.76, 5.91)	4.01 (2.75, 5.86)
Distant	26.30 (17.4, 39.76)	24.70 (16.47, 37.04)
**AGE, YEARS**		
30–39 (reference)	1.00	1.00
40–49	0.59 (0.33, 1.07)	0.58 (0.32, 1.04)
50–59	0.78 (0.46, 1.33)	0.77 (0.46, 1.30)
60–69	1.01 (0.59, 1.72)	0.99 (0.59, 1.68)
70+	1.25 (0.72, 2.16)	1.16 (0.67, 1.99)
**RACE**		
White (reference)	1.00	1.00
Black	1.08 (0.80, 1.46)	0.88 (0.62, 1.25)
**CDI**		
1 SD Increase	–	1.19 (1.01, 1.39)

*The event of interest is breast cancer-related death among triple negative breast cancer patients in Louisiana, diagnosed 2010–2012. HR, hazard ratio; CI, confidence interval; SEER, Surveillance, Epidemiology and End Results Program; CDI, concentrated disadvantage index; SD, standard deviation*.

*Model 1 is adjusted for SEER stage, age and race. Model 2 is adjusted for SEER stage, age, race and CDI*.

*Models include random intercept (frailty term) for US census tracts*.

a https://www.registrypartners.com/seer-summary-staging-manual-2000/

## Discussion

In the US, the incidence of TNBC in Black women is approximately 2-fold that of White women across all ages (6) and it has been shown to have the worst stage-specific survival of all breast cancer subtypes (32). The poor prognosis among TNBC patients is thought to be one of the major contributors to racial disparities in breast cancer mortality. While some investigators attribute it to differences in income and social status, which affect access to and compliance with treatment at disproportionate rates among minorities, others credit it to racial/ethnic differences in tumor biology and responsiveness to treatment (33–38). Our study supports the views expressed by other groups that both tumor biology and socioeconomic factors are major driving forces behind racial disparities, but their impact varies along the continuum of the disease. We found that CDI was not associated with disparities in incidence of TNBC but was associated with diagnosis at more advanced stages and poorer survival.

The role of social determinants in TNBC is of interest because low socioeconomic status is associated with many of the shared characteristics of breast tumors that occur in AA women, including high grade, high clinical stage at diagnosis, and ER-negative status (39, 40). In the US, socioeconomic status is intrinsically linked with race and lifestyle behaviors, such as physical activity, obesity, diet, reproductive experiences such as having more children, and screening behaviors, which vary in prevalence across different populations of women (41). The poverty rate in Louisiana is one of the highest in the nation, with socioeconomic conditions worse among AA. One in three Black Louisianans lives below the federal poverty level and has limited access to the health system (42). This study assessed the effects of neighborhood CDI, which is a robust measure of neighborhood environment. Our results support the notion that adverse living environments correlates with unfavorable stage and survival outcomes. It is therefore likely that socioeconomic disadvantages that are more prevalent in the AA community coupled with the increased risk for TNBC found for AA contribute to the high breast cancer mortality gap in Louisiana.

In view of the higher incidence of TNBC in women of African than of European ancestry, African ancestry might be associated with inherited genetic variants that predispose carriers to TNBC. One potential contributor to the higher incidence of early-onset aggressive breast cancer among African American patients may be a previously undefined higher burden of inherited breast cancer in this population. International studies have revealed that breast cancer frequencies of TNBC compared to other populations in the world (43). Triple negative breast cancer is known to be a marker of hereditary breast cancer susceptibility syndromes, such as BRCA1 mutations. High frequencies of mutations in *BRCA1* and *BRCA2* have already been observed in breast cancer patients of African ancestry from Nigeria (44) and from the Bahamas (45).

Our results indicate that disparities in neighborhood environment do contribute to racial disparities in TNBC outcomes between AA and EA in stage and survival, but not incidence. It is important to note that we lacked the case numbers needed to address disparities among other racial/ethnic groups. Additional limitations include a relatively limited study time period and cross-sectional design. SEER registries began collecting HER2 status in 2010; therefore only 3 years of data were available at the time of the study. Despite these limitations, we were able to identify indications of significant neighborhood level effects of concentrated disadvantage in these analyses. Due to the cross-sectional design, the duration of exposure or the risk associated with neighborhood environment over time was not established. An additional limitation is that we have assessed the effects of neighborhood living environment based on a census-defined spatial unit (tract), which is designed to be relatively homogeneous in terms of social characteristics but does lack a subjective definition of “neighborhood.” This study does not attempt to characterize other potentially influential social determinants of health, such as individual income or education. Finally, the study lacked information on individual level risk factors such as family history, reproductive history, comorbidities and health behaviors, which are needed to fully specify individual risk of this disease. The models did not adjust for cancer treatment, which also plays an important role in survival.

We have presented results from a population-based study in a single state regarding the impact of neighborhood concentrated disadvantage on disparities in TNBC outcomes. Our results support the need for a multipronged approach to address racial disparities that originate from the environment in which people live. The incidence of TNBC appears to be largely biologically determined, thus disparities will likely persist in terms of individual risk and incidence. However, disparities in TNBC outcomes are not due to biology alone, and these results highlight the need for targeted interventions and effective therapies among high-risk populations. Future efforts in breast cancer health equity should have a particular focus on individuals identified through known genetic risk and AA women in general.

## Author Contributions

FH and DD contributed to the study concept, design, methods, and contributed to manuscript development. OP and AG made considerable contributions of intellectual content and contributed to manuscript development. TF, NS, CL, and QY contributed to the study concept and revised the article for intellectual content. X-CW contributed to documentation of data and methods and revised the article for intellectual content. LM and RS made substantial contributions to the study concept, design and intellectual content. All authors have approved the content and agree to be accountable for the work with regard to accuracy and integrity.

### Conflict of Interest Statement

The authors declare that the research was conducted in the absence of any commercial or financial relationships that could be construed as a potential conflict of interest.

## Footnotes

^1^NAACCR Geocoder https://www.naaccr.org/gis-resources/

^2^PhenX Toolkit – Neighborhood Concentrated Disadvantage Index https://www.phenxtoolkit.org/index.php?pageLink=browse.protocoldetails&id=211302

^3^American FactFinder https://factfinder.census.gov/faces/nav/jsf/pages/index.xhtml

^4^US Census Geographic Areas Reference Manual https://www.census.gov/geo/reference/garm.html

^5^SEER Summary Staging Manual 2000 https://seer.cancer.gov/tools/ssm/ssm2000/

^6^SEER Cause of Death https://seer.cancer.gov/codrecode
