# Supplementary material for: Neighborhood Social Determinants of Triple Negative Breast Cancer
Source: Front Public Health. 2019 Feb 18;7:18. doi: 10.3389/fpubh.2019.00018 (PMC6387917; doi:10.3389/fpubh.2019.00018)
Supplement: Supplementary file 1 [file Table_1.docx]

**Supplemental Table S1.**Factor loadings for individual census tract measures used in the principal components analysis for concentrated disadvantage index (CDI), Louisiana 2008-2012.

| Variable | Factor  Loading |
| --- | --- |
| Poverty | 0.83 |
| Public Assistance | 0.61 |
| Female-headed Families | 0.84 |
| Unemployed | 0.77 |
| Under 18 | 0.43 |
| Black | 0.86 |
